# Tumor growth activity of duloxetine in Ehrlich carcinoma in mice

**DOI:** 10.1186/s13104-018-3655-4

**Published:** 2018-07-31

**Authors:** Ed Carlos Rey Moura, Plinio da Cunha Leal, Izabel Cristina Portela Bogéa Serra, Bruno de Paulo Ribeiro, Johnny Ramos do Nascimento, Flavia Raquel Fernandes do Nascimento, Rioko Kimiko Sakata

**Affiliations:** 1 Universidade Federal do Maranhão, São Luís, Brazil; 2 Universidade Federal de São Paulo, Rua Três de Maio 61/51, Vila Clementino, São Paulo, Brazil

**Keywords:** Cancer, Duloxetine, Tumor growth

## Abstract

**Objective:**

The objective of this study was to analyze whether duloxetine influences tumor growth in Ehrlich carcinoma. The mice were administered 5 or 30 mg/kg of duloxetine or saline solution. All animals were inoculated with tumor cells. The tumor progression was evaluated by body weight, abdominal circumference, ascites volume and tumor cell count. The effect of duloxetine on immune response was evaluated by lymphoid cells, nitric oxide (NO) production, arginase and superoxide dismutase (SOD) activity and the spleen immunophenotyping.

**Results:**

There was no difference between the groups regarding weight, abdominal circumference, ascites volume and number of tumor cells. Duloxetine increased the cells of the inguinal lymph node. There was no difference in the number of cells in the bone marrow and spleen. Ascites SOD activity was greater in Duloxetine groups. There were no differences in the levels of NO, nitrite, and arginase. The number of antibody for CD3 (CD3+), CD4+, CD8+ and CD28+ cells was lower in the duloxetine groups. In conclusion, duloxetine has no direct effect on tumor growth and does not alter immunity. The drug increased the SOD that fights free radicals and led the migration of lymphocytes, suggesting that duloxetine could be used in tumor-bearing individuals.

**Electronic supplementary material:**

The online version of this article (10.1186/s13104-018-3655-4) contains supplementary material, which is available to authorized users.

## Introduction

Cancer pain is treated according to the analgesic ladder, with opioids on the second and third steps. Adjuvants, such as duloxetine, can be used in combination on all steps. The use of antidepressants is important to appropriately treat cancer pain. The study on the effect of adjuvants is important to select drugs that do not increase tumor growth.

In the Ehrlich’s tumor, a mammary carcinoma of female mice in ascitic form, pain is caused by the inflammatory response, and pressure exerted by progressive tumor enlargement, hemorrhage, and neural compression [1].

Duloxetine would be an interesting analgesic drug for its anti-inflammatory effect [2]. Duloxetine is a reuptake inhibitor for serotonin (5-HT) and norepinephrine in the central nervous system that reduces pain transmission [3]. Duloxetine, in vitro, was able to decrease the viability of hepatocellular carcinoma cells [4].

There aren’t any studies about the in vivo effect of duloxetine on the effect of duloxetine on the tumor progression and on the immune response.

The present study aimed to evaluate whether duloxetine directly influences tumor growth, in the Ehrlich’s tumor model in mice.

## Main text

### Materials and methods

After approval from the Ethics Committee (CEUA 5923020415), 15 adult female Swiss mice, with 60 days and between 25 and 30 g were housed 5/cage on a 12-h-light/-dark cycle in a temperature-controlled room with food and water available ad libitum. Animal experiments were carried out in accordance with the International Association for the Study of Pain (IASP). Every effort was made to reduce the number of animals used and their discomfort.

The mice were divided into three groups with 5 animals each. Groups D5 and D30 received 5 and 30 mg/kg of duloxetine, respectively; group S (control) received 0.2% of hydroxypropyl methylcellulose. On the first day, all animals received an intraperitoneal inoculation of 2 × 10^6^ Ehrlich ascites carcinoma cells. From the second to the eighth day, duloxetine in 0.2% of hydroxypropyl methylcellulose or 0.2% hydroxypropyl methylcellulose was administered daily by gavage. All animals received the same volume (10 mL/kg) and concentration of the vehicle. The animals were euthanized by lethal injection of 100 μL of anesthetic drugs (2:1 solution of 10% ketamine and 20% xilasine hydrochloride, 100 uL) by intramuscular route.

Ehrlich’s tumor is maintained in the Immunophysiology laboratory of UFMA, through weekly intraperitoneal transplantation of tumor cells (2 × 10^6^/animal) in healthy Swiss mice. To obtain the tumor cells, mice with approximately 7-day evolution of the Ehrlich ascites tumor are sacrificed, the ascites fluid collected from the peritoneal cavity, resuspended in centrifuged phosphate buffered solution and the cells are resuspended in 5 mL of phosphate buffered saline (PBS). To determine the concentration of tumor cells presents in this suspension, cells are counted in the Neubauer chamber, and cells viability is determined by the trypan blue exclusion test. Only suspensions with viability greater than 95% were used.

These were evaluated: weight and abdominal circumference both measured on the first and tenth day; volume of ascites; viable ascites tumor cells; number of cells of lymphoid organs (femur, spleen and inguinal lymph nodes), by optical microscopy; NO in the ascites and spleen, determined by nitrites, measured by colorimetric assay; arginase in ascites; SOD in ascites; and immunophenotyping of spleen.

The results were analyzed using Graph Pad Prism 5.0^®^. There was used the Kruskal–Wallis test to compare the groups. The level of significance considered was p ≤ 0.05.

### Results

The treatment with Duloxetine has no effect on tumor development. There was no difference between the groups regarding weight, abdominal circumference, ascites volume and number of tumor cells (Table 1). Leukocyte concentration in the inguinal lymph node was higher in Duloxetine groups. The treatment with duloxetine had no effect on other lymphoid organs; there was no difference in the number of cells in the bone marrow and spleen (Table 1).

**Table 1:** Weight, volume of ascites, abdominal circumference, tumor cells, and leukocytes (median/IQR)

	D5	D30	S	p
Weight (g)	41 (37.5–41.5)	41 (34–44)	34 (32.5–39.5)	0.160
Ascites V (mL)	8.2 (5–8.6)	8 (6.0–8.3)	6.2 (3.3–7.1)	0.140
Circumference (cm)	10 (9.5–10.2)	10.5 (9.7–11.2)	9.5 (8.5–10.4)	0.216
Living tumor cel. (× 10^6^/mL)	390 (272–502)	375 (280–607)	472 (265–516)	0.887
Leukocytes (× 10^8^/mL) Spleen	2500 (1813–3313)	2000 (1250–2375)	2300 (1325–3700)	0.509
IL	240 (167–682)*	135 (130–190)*	85 (65–142)	0.015
BN	600 (412–815)	995 (617–1005)	510 (440–667)	0.112

*D5* 5 mg/kg, *D30* duloxetine 30 mg/kg, *S* saline, *V* volume, *Cel* cells, *ML* mesenteric lymph nodes, *IL* inguinal lymph node, *BN* bone narrow, Kruskal–Wallis test, *IQR* interquartile

*Significant

Both Duloxetine groups increased SOD activity in ascites (Table 2).There were no differences in the levels of NO, nitrite and arginase between groups (Table 2).

**Table 2:** Effect of Duloxetine treatment on concentration of NO, nitrite, arginase and SOD activity from Ehrlich tumor-bearing mice

	D5	D30	C	p
Spleen NO (mM)	3.5 (2.5–4.5)	6.4 (3.1–10.0)	1.6 (0.8–4.0)	0.080
Ascites NO (mM)	13.9 (11.4–16.7)	12.7 (7.0–19.6)	12.6 (11.2–18.3)	0.801
Ascites nitrite (mM)	5.1 (4.9–7.1)	5.0 (4.6–5.6)	5.4 (4.9–5.6)	0.499
Ascites arginase (unit/L)	48.6 (45.8–51.4)	52.9 (45.5–56.7)	48.1 (36.7–67.7)	0.690
Ascites SOD (% activity)	92.5 (88.9–96.9)*	91.6 (87.7–94.2)*	87.6 (86.5–88.4)	0.050

The animals were treated with duloxetine (5 and 30 mg/kg) or saline (control) orally from days 2 to 8 after intraperitoneal inoculation of the 2 × 10^6^ Ehrlich tumor cells (median/IQR)

*D5* 5 mg/kg, *D30* duloxetine 30 mg/kg; *S* saline, *NO* nitric oxide, *SOD* superoxide dismutase, Kruskal–Wallis test, *IQR* interquartile

*Significant

Duloxetine treatment decreased T lymphocyte number and its activation; the absolute number of CD3+ lymphocytes was lower in duloxetine groups. There was no difference in the absolute frequency of activated CD4+ and CD8+ lymphocytes between groups. Finally, the expression of the costimulatory molecule CD28 in both T helper lymphocyte (T CD4) and cytotoxic T lymphocyte (T CD8) lymphocytes was lower in the Duloxetine groups (Table 3).

**Table 3:** Immunophenotyping of spleen (median/IQR)

	D5	D30	S	p
CD3 (× 10^7^/mL)^a^	1.0 (0.7–1.4)*	1.3 (1.1–1.6)*	2.2 (1.2–2.4)	0.045
CD4 (× 10^7^/mL)^a^	0.7 (0.5–0.9)	0.8 (0.6–0.9)	1.5 (1.0–1.7)	0.2448
CD8 (× 10^7^/mL)^a^	0.2 (0.1–0.2)	0.3 (0.2–0.5)	0.4 (0.2–0.5)	0.088
CD28 (× 10^5^/mL)^b^	39 (37–40)*	42 (39–45)*	51 (45–52)	0.010

*D5* 5 mg/kg, *D30* duloxetine 30 mg/kg, *S* saline

^a^Absolute frequency of spleen lymphocytes

^b^Fluorescence intensity of spleen lymphocytes; Kruskal–Wallis test; *IQR* interquartile

*Significant

There was listed the information about Additional material, with file name, title of data and description of data at the section Additional file 1.

### Discussion

In this study, it was found that duloxetine does not show a harmful role in tumor growth and proliferation, but it caused a significant reduction in the number of spleen T lymphocytes, and in its activation, as evidenced by immunophenotyping, as well as increased SOD activity, suggesting a reduction in the inflammatory response in the tumor-bearing mice.

The action of duloxetine on tumor growth was studied given that it is important to use adjuvants that do not exacerbate tumor growth. Adjuvants with fewer negative effects on tumor dissemination should be chosen, and one study suggested that duloxetine inhibits tumor growth [4]. Duloxetine in doses of 5 and 30 mg/kg was administered based on the experimental literature [5, 6].

Pain results in the release of cytokines and reduces immunity, so pain control is extremely relevant. Good pain control results in a reduction in the tumor metastasis [7].

Tumor inoculation was similar in the Fernandes et al. [8] with the same number of tumor cells as in Patra et al. [9].

Immunophenotyping of the spleen was performed given that it is a site of great accumulation of defense cells. NO is degraded generating nitrite, so nitrite indirectly measures NO production [10]. Duloxetine reduced, though not significantly, the concentration of nitrite. The role of NO is twofold: it promotes tumor dissemination and is also tumoricidal [11]. At lower concentrations (< 100 nM), NO causes tumor progression [11] and at higher concentrations (> 100 nM), it induces apoptosis [12]. NO can promote tumor growth at the concentration measured. Arginine metabolism is important for reducing cell death [13]. Increased arginase activity, related to tumor growth, decreases the production of NO [14].

Tumor growth is associated with the reduction of SOD activity [9, 12]). SOD activity increased with duloxetine as well as in another study [15]. SOD protects cells from damage [16].

After assessing the tumor microenvironment (dosage of NO, arginase, nitrite and SOD) we decided to evaluate whether duloxetine influences the immune system. Therefore, we evaluated their cellular impact, quantifying the T lymphocytes in the spleen.

The number of T lymphocytes stained with the CD3 antibody (CD4+ T and CD8+ T cells) was lower with duloxetine. CD4+ and CD8+ T lymphocytes destroy tumor cells [17]. In addition, there was a lower number of CD4+ and CD8+ T cells expressing the CD28 receptor with duloxetine. CD28 hinders cellular energy production [18].

As the cells were activated and had a decrease in the spleen it was assumed that the lymphocytes, when activated, migrated from the spleen to other sites, such as inguinal lymph nodes. Hofstetter et al. [19] also observed this effect on lymphocytes when they studied the influence of 5-HT reuptake inhibitor drugs on the immunity.

In conclusion, duloxetine has no direct effect on tumor growth and does not alter immunity. The drug increased the SOD that fights free radicals and led to the migration of lymphocytes, such as other drugs of its group. Due to its analgesic effect, Duloxetine can be regarded as an adjuvant option for cancer pain management.

## Limitations

The results obtained in animals cannot be directly generalized to humans, and the results may vary with different tumor types. Future studies may show whether the data can be extrapolated to humans. However, as aforementioned, this is the first study investigating whether this drug may worsen tumors.

## Additional file

**Additional file 1.** Additional tables.

## Abbreviations

5-HT: serotonin
CD3: antibody for CD3
D: duloxetine
IASP: International Association for the Study of Pain
NO: nitric oxide
PBS: phosphate buffered saline
SOD: superoxide dismutase
T CD4+: T helper lymphocyte
T CD8+: cytotoxic T lymphocyte
UFMA: Universidade Federal do Maranhão
