# Supplementary material for: Tumor growth activity of duloxetine in Ehrlich carcinoma in mice
Source: BMC Res Notes. 2018 Jul 31;11:525. doi: 10.1186/s13104-018-3655-4 (PMC6069801; doi:10.1186/s13104-018-3655-4)
Supplement: Supplementary file 1 — Additional file 1. Additional tables. [file 13104_2018_3655_MOESM1_ESM.doc]

**Additional material**

**File 1. Effect of duloxetine treatment on Survival (days).** The animals were treated with duloxetine (D) 5 and 30 mg/kg, P.O. or Saline (C) control, P.O., from days 1 to 8 after inoculum (2x10^6^ /animal, ip.) of the Ehrlich tumor. The animals were sacrficed on the tenth day.

| Animals | D5 | D30 | C |
| --- | --- | --- | --- |
| 1 | 14 | 16 | 11 |
| 2 | 11 | 21 | 12 |
| 3 | 12 | 11 | 18 |
| 4 | 15 | 17 | 16 |
| 5 | 17 | 19 | 19 |

**File 2. Effect of duloxetine treatment on Weight (grams). The animals were treated with duloxetine (D) 5 and 30 mg / kg, P.O. or Saline (C) control, P.O., from days 1 to 8 after inoculum (2x10^6^ / animal, ip.) of the Ehrlich tumor. The animals were euthanized on the tenth day.**

| Animals | D5 | D30 | C |
| --- | --- | --- | --- |
| 1 | 42 | 30 | 34 |
| 2 | 35 | 44 | 39 |
| 3 | 41 | 41 | 31 |
| 4 | 40 | 44 | 34 |
| 5 | 41 | 38 | 40 |

**File 3. Effect of duloxetine treatment on Abdominal circumference (cm).** The animals were treated with duloxetine (D) 5 and 30 mg / kg, P.O. or Saline (C) control, P.O., from days 1 to 8 after inoculum (2x10^6^ / animal, ip.) of the Ehrlich tumor. The animals were euthanized on the tenth day.

| Animals | D5 | D30 | C |
| --- | --- | --- | --- |
| 1 | 10,5 | 10 | 10,8 |
| 2 | 10 | 9,5 | 9 |
| 3 | 9 | 11 | 9,5 |
| 4 | 10 | 10,5 | 8 |
| 5 | 10 | 11,5 | 10 |

**File 4. Effect of duloxetine treatment on Ascites volume (ml). The animals were treated with duloxetine (D) 5 and 30 mg / kg, P.O. or Saline (C) control, P.O., from days 1 to 8 after inoculum (2x10^6^ / animal, ip.) of the Ehrlich tumor. The animals were euthanized on the tenth day.**

| Animals | D5 | D30 | C |
| --- | --- | --- | --- |
| 1 | 4,3 | 7,8 | 6,7 |
| 2 | 8,2 | 4,2 | 6,2 |
| 3 | 7,5 | 8,2 | 6,1 |
| 4 | 8,4 | 8,4 | 0,6 |
| 5 | 8,9 | 8 | 7,6 |

**File 5. Effect of duloxetine treatment on Total tumor cells (x10^7^). The animals were treated with duloxetine (D) 5 and 30 mg / kg, P.O. or Saline (C) control, P.O., from days 1 to 8 after inoculum (2x10^6^ / animal, ip.) of the Ehrlich tumor. The animals were euthanized on the tenth day.**

| Animal | D5 | D30 | C |
| --- | --- | --- | --- |
| 1 | 565 | 410 | 489 |
| 2 | 468 | 363 | 572 |
| 3 | 324 | 622 | 508 |
| 4 | 363 | 665 | 98 |
| 5 | 438 | 240 | 472 |

**File 6. Effect of duloxetine treatment on Leukocytes in the spleen (x 10^4^/mL).** The animals were treated with duloxetine (D) 5 and 30 mg / kg, P.O. or Saline (C) control, P.O. from days 1 to 8 after inoculum (2x10^6^ / animal, ip.) of the Ehrlich tumor. The animals were euthanized on the tenth day.

| Animals | D5 | D30 | C |
| --- | --- | --- | --- |
| 1 | 2625 | 500 | 3175 |
| 2 | 2125 | 2750 | 1125 |
| 3 | 2500 | 2000 | 2300 |
| 4 | 1500 | 2000 | 4225 |
| 5 | 4000 | 2000 | 1475 |

**File 7. Effect of duloxetine treatment on Leukocytes in inguinal lymph node (x 10^4^/mL).** The animals were treated with duloxetine (D) 5 and 30 mg / kg, P.O. or Saline (C) control, P.O., from days 1 to 8 after inoculum (2x10^6^ / animal, ip.) of the Ehrlich tumor. The animals were euthanized on the tenth day.

| Animals | D5 | D30 | C |
| --- | --- | --- | --- |
| 1 | 140 | 135 | 110 |
| 2 | 195 | 170 | 55 |
| 3 | 1050 | 130 | 75 |
| 4 | 240 | 130 | 175 |
| 5 | 315 | 210 | 85 |

**File 8. Effect of duloxetine treatment on Leukocytes in the bone marrow (x 10^3^/mL).** The animals were treated with duloxetine (D) 5 and 30 mg / kg, P.O. or Saline (C) control, P.O., from days 1 to 8 after inoculum (2x10^6^ / animal, ip.) of the Ehrlich tumor. The animals were euthanized on the tenth day.

| Animals | D5 | D30 | C |
| --- | --- | --- | --- |
| 1 | 855 | 1015 | 410 |
| 2 | 475 | 760 | 610 |
| 3 | 350 | 475 | 510 |
| 4 | 600 | 995 | 725 |
| 5 | 775 | 995 | 470 |

**File 9. Effect of duloxetine treatment on Spleen NO (mM). The animals were treated with duloxetine (D) 5 and 30 mg / kg, P.O. or Saline (C) control, P.O., from days 1 to 8 after inoculum (2x10^6^ / animal, ip.) of the Ehrlich tumor. The animals were euthanized on the tenth day.**

| Animal | D5 | D30 | C |
| --- | --- | --- | --- |
| 1 | 2,59 | 2,94 | 5,6 |
| 2 | 4,51 | 10,33 | 2,45 |
| 3 | 2,59 | 3,85 | 0,90 |
| 4 | 4,51 | 9,06 | 1,63 |
| 5 | 4,00 | 9,00 | 0,76 |

**File 10. Effect of duloxetine treatment on Ascites NO (mM). The animals were treated with duloxetine (D) 5 and 30 mg / kg, P.O. or Saline (C) control, P.O., from days 1 to 8 after inoculum (2x10^6^ / animal, ip.) of the Ehrlich tumor. The animals were euthanized on the tenth day.**

| Animal | D5 | D30 | C |
| --- | --- | --- | --- |
| 1 | 11,26 | 9,24 | 9,02 |
| 2 | 11,82 | 20,86 | 17,56 |
| 3 | 16,02 | 5,24 | 12,52 |
| 4 | 16,98 | 18,04 | 11,18 |
| 5 | 17,64 | 8,32 | 20,24 |

**File 11. Effect of duloxetine treatment on Ascites Nitrite (mM). The animals were treated with duloxetine (D) 5 and 30 mg / kg, P.O. or Saline (C) control, P.O., from days 1 to 8 after inoculum (2x10^6^ / animal, ip.) of the Ehrlich tumor. The animals were euthanized on the tenth day.**

| Animals | D5 | D30 | C |
| --- | --- | --- | --- |
| 1 | 4,47 | 8,19 | 5,48 |
| 2 | 4,81 | 5,12 | 5,60 |
| 3 | 5,88 | 4,87 | 5,75 |
| 4 | 5,01 | 5,19 | 4,58 |
| 5 | 5,37 | 6,07 | 5,37 |

**File 12. Effect of duloxetine treatment on Ascites Arginase (und/L). The animals were treated with duloxetine (D) 5 and 30 mg / kg, P.O. or Saline (C) control, P.O., from days 1 to 8 after inoculum (2x10^6^ / animal, ip.) of the Ehrlich tumor. The animals were euthanized on the tenth day.**

| Animals | D5 | D30 | C |
| --- | --- | --- | --- |
| 1 | 53,70 | 50,18 | 38,74 |
| 2 | 49,21 | 58,13 | 63,34 |
| 3 | 48,28 | 40,93 | 34,71 |
| 4 | 48,61 | 52,90 | 72,14 |
| 5 | 43,30 | 23,85 | 48,16 |

**File 13. Effect of duloxetine treatment on Ascites SOD (%Activity). The animals were treated with duloxetine (D) 5 and 30 mg / kg, P.O. or Saline (C) control, P.O., from days 1 to 8 after inoculum (2x10^6^ / animal, ip.) of the Ehrlich tumor. The animals were euthanized on the tenth day.**

| Animals | D5 | D30 | C |
| --- | --- | --- | --- |
| 1 | 88,48 | 96,01 | 87,67 |
| 2 | 89,38 | 84,62 | 87,00 |
| 3 | 92,53 | 92,46 | 87,63 |
| 4 | 98,24 | 90,95 | 89,15 |
| 5 | 95,73 | 91,65 | 86,15 |

**File 14. Effect of duloxetine treatment on CD3 (x 10^6^). The animals were treated with duloxetine (D) 5 and 30 mg / kg, P.O. or Saline (C) control, P.O., from days 1 to 8 after inoculum (2x10^6^ / animal, ip.) of the Ehrlich tumor. The animals were euthanized on the tenth day.**

| Animals | D5 | D30 | C |
| --- | --- | --- | --- |
| 1 | 1,98 | 2,49 | 2,60 |
| 2 | 1,84 | 2,84 | 2,42 |
| 3 | 1,98 | 1,80 | 2,02 |
| 4 | 1,84 | 1,68 | 3,44 |
| 5 | 1,95 | 1,78 | 3,47 |

**File 15. Effect of duloxetine treatment on CD4 (x 10^7^). The animals were treated with duloxetine (D) 5 and 30 mg / kg, P.O. or Saline (C) control, P.O., from days 1 to 8 after inoculum (2x10^6^ / animal, ip.) of the Ehrlich tumor. The animals were euthanized on the tenth day.**

| Animals | D5 | D30 | C |
| --- | --- | --- | --- |
| 1 | 0,070 | 0,279 | 0,224 |
| 2 | 0,154 | 0,195 | 0,245 |
| 3 | 0,070 | 0,162 | 0,275 |
| 4 | 0,154 | 0,157 | 0,382 |
| 5 | 0,090 | 0,160 | 0,393 |

**File 16. Effect of duloxetine treatment on CD8 (x 10^7^). The animals were treated with duloxetine (D) 5 and 30 mg / kg, P.O. or Saline (C) control, P.O., from days 1 to 8 after inoculum (2x10^6^ / animal, ip.) of the Ehrlich tumor. The animals were euthanized on the tenth day.**

| Animals | D5 | D30 | C |
| --- | --- | --- | --- |
| 1 | 0,151 | 0,535 | 0,4550 |
| 2 | 0,281 | 0,395 | 0,2447 |
| 3 | 0,151 | 0,332 | 0,3210 |
| 4 | 0,281 | 0,264 | 0,5630 |
| 5 | 0,250 | 0,260 | 0,5970 |

**File 17. Effect of duloxetine treatment on CD28 (x 10^5^). The animals were treated with duloxetine (D) 5 and 30 mg / kg, P.O. or Saline (C) control, P.O., from days 1 to 8 after inoculum (2x10^6^ / animal, ip.) of the Ehrlich tumor. The animals were euthanized on the tenth day.**

| Animals | D5 | D30 | C |
| --- | --- | --- | --- |
| 1 | 37,4 | 44,1 | 44,2 |
| 2 | 40,6 | 39,0 | 53,9 |
| 3 | 37,4 | 45,8 | 51,2 |
| 4 | 40,6 | 41,1 | 51,9 |
| 5 | 39,0 | 40,0 | 46,8 |
